# Effect of Floor Cooling on Behavior and Heart Rate of Late Lactation Sows Under Acute Heat Stress

**DOI:** 10.3389/fvets.2018.00223

**Published:** 2018-09-21

**Authors:** Severine P. Parois, Francisco A. Cabezón, Allan P. Schinckel, Jay S. Johnson, Robert M. Stwalley, Jeremy N. Marchant-Forde

**Affiliations:** ^1^PEGASE, Agrocampus Ouest, INRA, Saint-Gilles, France; ^2^Department of Animal Sciences, Purdue University, West Lafayette, IN, United States; ^3^Livestock Behavior Research Unit, USDA-ARS, West Lafayette, IN, United States; ^4^Department of Agricultural and Biological Engineering, Purdue University, West Lafayette, IN, United States

**Keywords:** sow, cooling pads, heat stress, lactation, behavior, heart rate

## Abstract

Much U.S. swine production is in Köppen climate types classified as “hot-summer humid continental” and “humid subtropical.” As a result, farrowing sows are often exposed to temperatures above their upper critical temperature. This heat stress (HS) can affect sow welfare and productivity and have a negative economic impact. The study objective was to evaluate the impact of a cooling pad on sows' behavioral and heart rate responses to acute HS. Treatments were randomly allotted to ten multiparous sows to receive a constant cool water flow of 0.00 (CONTROL, *n* = 4), 0.25 (LOW, *n* = 2), 0.55 (MEDIUM, *n* = 2), or 0.85 (HIGH, *n* = 2) L/min for 100 min and replicated eight times, switching treatments so that each sow was exposed to each treatment. The cooling was initiated 1 h after the room reached 35°C for 100 min. Eating, drinking and nursing behaviors, postures, and heart rate were recorded before heating (Period 1), prior to cooling (Period 2), and during cooling (Period 3). There were no differences between LOW, MEDIUM, and HIGH flow rates for any periods on all behavioral and heart rate traits, so data were pooled (COOLED). There were no differences in any of the measures during Periods 1 and 2, except for the ratio of short term to long term heart rate variability (SD1:SD2) with higher values for CONTROL than COOLED sows in Period 2. During Period 3, CONTROL sows changed postures more frequently (11.5 ±1.6 vs. 5.1 ±1.6 changes per hour), spent more time drinker-pressing/drinking (4.4 ± 0.5 vs. 1.4 ± 0.4% of time), standing (6.6 ± 1.7 vs. 3.8 ± 1.6% of time), sitting (10.0 ± 1.2 vs. 4.0 ± 1.1), less time lying (83.0 ±1.8 vs. 92.0 ±1.7% of time), especially lying laterally (62.0 ± 5.6 vs. 75.0 ± 5.3% of time), than sows in all three cooling treatments (all P < 0.001). Heart rate during Period 3 was lower for COOLED sows compared to the CONTROL sows (100.2 ± 3.4 vs. 119.0 ± 4.0 beat per min, P < 0.001). Sows response to increased thermal load can be effectively reduced using water-cooled cooling pads, thereby improving sow comfort and welfare. The beneficial effects on behavior are noticeable from the lowest flow rate.

## Introduction

Pork continues to be the world's most-consumed meat (1), and the majority of global pork production is carried out in areas that are subject to thermal extremes. The amount of animal production subject to thermal extremes is increasing due to the combined impacts of climate change (2), human population increase, and increased demand for animal protein in developing countries (3). Against this background, for demand to be met, there needs to be improvements in production efficiency and environmental sustainability (4).

Heat stress conditions in pigs can be described with two concepts: the preferred temperature range, which is the optimal range, and the upper extreme temperature, which can cause serious negative effects in terms of performance and welfare. The preferred temperature range in pigs decreases with age from a minimum of 32°C before 3 kg, 26–32°C in prenursery stage 10–25°C for growing/finishing pigs and sows or boars above 100 kg (5, 6). Lactating sows have an optimal range between 15 and 26°C. The upper critical temperature for piglets from 3 kg to finishing pigs is 35°C, whereas it is 32°C for lactating and sows or boars above 100 kg (6).

Extreme heat impacts both pig production and pig welfare in multiple ways (7, 8). Heat stressed pigs show increases in body temperatures, which they attempt to counteract by increasing respiration rates (5) and altering behavior, reducing activity and lying laterally (9) to promote heat loss, as well as increasing wallowing (10) and shade seeking when housed outside (11). They reduce feed intake, resulting in depressed growth rates in growing pigs and loss of body condition (5) and reduced milk production in lactating sows (12, 13), with the knock-on effect on piglet growth (14). There can be a reduction in gastro-intestinal health (15) and changes in carcass composition, with an increase in fat deposition (16). For sows, exposure to heat stress at specific periods during the reproductive cycle can result in anestrus, decreased farrowing rates, increased embryonic mortality, and decreased litter sizes (17). Gestation length can be reduced (18), but farrowing duration increased (9), increasing risk of stillbirths. As well as the direct effect on offspring as a consequence of altered milk production, offspring from heat stressed mothers can themselves have lower fertility, with gilts having smaller litters (19) and boars having poorer sperm quality (20). Overall, the economic cost is in the hundreds of millions of dollars annually, in the USA alone (21).

In order to counteract the impact of heat stress, different cooling methods have been developed. Options include whole building cooling systems and systems that target the individual pig, using one or more of convection, conduction, and evaporation (22). Evaporative cooling systems are most often used with drip or sprinkler systems (23, 24), often combined with mechanical ventilation. Another option is air cooling directed specifically at the head region—snout cooling (25). While these systems demonstrate some benefits, their effectiveness may be limited when there is high humidity in the case of evaporative systems (26), or by applicability only to pigs housed in close confinement such as sows in farrowing crates. There has also been some interest in conductive cooling such as that offered by cooled flooring (23, 27–29), and preference studies have shown that this type of cooling may be preferred by the pigs as it is close to their natural cooling behaviors (23).

The modern lactating sow is especially at risk of heat stress, as it has been heavily selected for increased productivity including litter size and litter weaning weight and thus resulting in increased heat production in comparison to past sows (30). Given that sows within a farrowing room may be at different stages of lactation, have different litter sizes and be producing heat unequally, individual sow cooling has the possibility to confer more production and welfare benefits than whole room cooling. Recently, a cooling pad has been designed to increase the potential removal of excess heat of modern lactating sows in high environmental temperatures (31). It showed beneficial effects on the sows' respiration rates, and vaginal, skin, and rectal temperatures (32), with a “dose-dependent” decrease for all traits with increasing water flow rates from LOW to HIGH (from 0.25 to 0.85 L/min for 100 min) in comparison to a CONTROL flow rate (0.00 L/min). The ultimate goal is that the system will be controllable at the individual sow level, potentially sampling welfare parameters from the sow in real time and using that information to adjust the cooling efficiency of the system. In order to reach this ultimate goal, there is a need to obtain fundamental information about the sows' behavioral and physiological responses to heat stress and how these can be influenced. Therefore, the study objective was to evaluate the impact of different water flow rates through a cooling pad on the sows' behavioral and heart rate responses.

## Materials and methods

The study was approved by Purdue University Institutional Animal Care and Use Committee and received the authorization number #1508001275.

### Animals, housing, and husbandry

The experiment was conducted from July 22nd to July 26th, 2016, at the swine farrowing facility at Purdue University Animal Sciences Research farm. The farm is located in a humid continental climate with warm summers (40° 29' 59” N and 87° 00' 47” W, with an altitude of 218 m) and a Köppen climate classification of Dfa (33).

**Figure 1:**
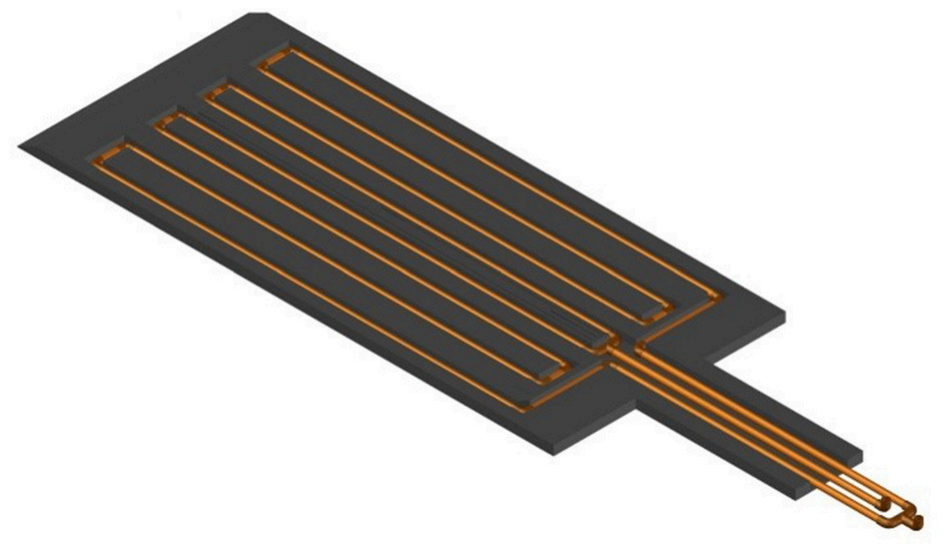
Cooling pad base and cooling water pipes [Extracted from Cabezón et al. (30).

The study subjects were 10 multiparous sows (commercial crossbred Yorkshire and Landrace), housed within the same farrowing room in individual farrowing crates (2.1 m × 0.6 m) within pens (2.3 m × 2.0 m), with fully-slatted metal floors. Each sow was provided with a cooling pad made with an aluminum diamond plate top, a high-density polyethylene base and eight copper water pipes in contact with the diamond plate through a specialty extruded aluminum clip (modified from 30, Figure 1). A more detailed description of the pad is available in the Patent Application (34). Each cooling pad had an outlet valve to regulate the water flow and an inlet valve to take inlet water samples. Piglets were provided supplementary heat using one heat lamp per farrowing crate, placed to one side over a solid polyethylene mat (1.0 m × 0.3 m).

The farrowing room had one heater and one fan that worked independently from each other. The heater was set to achieve 35°C once the trial started. The fan (fan and screen opening of 0.41 × 0.41 and 0.48 × 0.46 m, respectively) had 2 operating options (off or 100% speed). The fan was running at 100% speed during the entire trial. The screen opening in the fan was reduced to 50% in order to maintain the temperature. Temperature, relative humidity and dew point in the farrowing room were recorded in 5 min intervals, using 2 data loggers (accuracy: ± 0.5°C, 3% and 1.1°C for temperature, relative humidity and dew point, respectively, EL-USB-2, DATAQ Instruments, Inc., OH, USA). The data loggers were calibrated with a scientific thermometer and were placed 0.7 m from the floor at the sow level and away from water sources.

Sows were fed a corn and soybean meal-based diet with 5% distillers dried grains and solubles (DDGS) and 3% choice white grease. The diet was formulated to meet or exceed nutrient requirements (0.9% SID lysine, 19.9% CP, 3348 Kcal/kg ME and 2501 Kcal/kg NE) (NRC, 2012). Feedings occurred at 0700, 1,300, and 1,730 h each day to target *ad libitum* intake for each sow. All sows were fed a fixed amount (2.27 kg) before the morning (0700 h) and afternoon (1,300 h) repetitions of the experimental procedure. At the last feeding (1,730 h) sows were fed a variable amount of 1.81 kg, if the feeder was empty, or 1.36 kg, if there was feed left in the feeder. This method of feeding was to reduce the variation in heat production due to the amount and the time that the feed was consumed. Sows had *ad libitum* access to water.

Piglet processing (ear notching, tail docking, castration, teeth clipping and supplemental iron injection) was performed during the first 48 h post-partum. Piglet cross fostering was allowed only during the first 48 h post-partum (after processing), and litter size was standardized to ~10 or more piglets per sow (mean 11.2 ± 0.8).

### Experimental design

**Figure 2:**
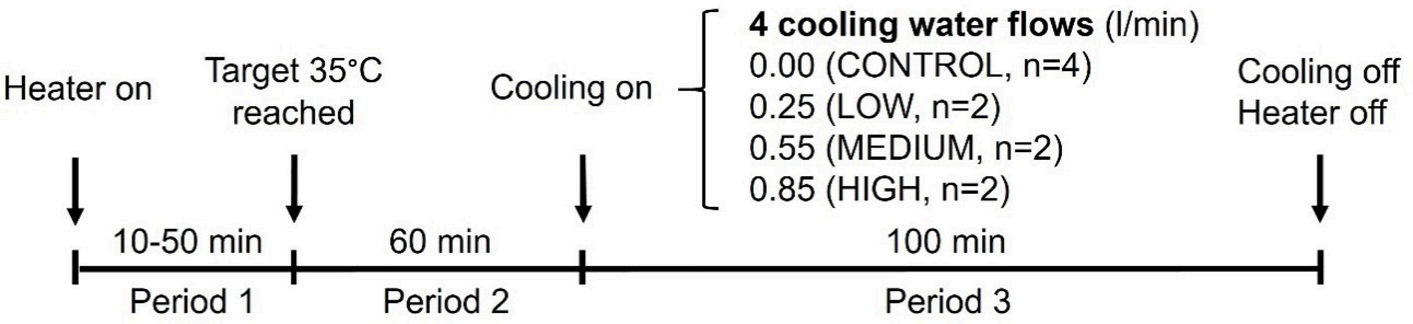
Protocol followed for each repetition of the trial. The protocol for the 10 sows was repeated 8 times (2 times/day for 4 days). The water flow rates assigned for each sow were calibrated before the trial. Behavior and heart rate were recorded continuously over the 3 periods.

The trial was conducted during late lactation when the average lactation length of the sows was 15.3 ± 2.8 days. A protocol outline of the trial is presented in Figure 2. Each trial consisted of three phases: Period 1—warming phase (variable time taken for room temperature to reach 35°C, from 10 to 50 min: 28.1 ± 12.2 min), Period 2—maintenance phase (1 h with temperature maintained at 35°C), and Period 3—cooling phase (100 min with temperature maintained at 35°C with cooling treatment applied). Treatments were randomly allotted to sows to receive a constant cool water flow of 0.00 (CONTROL, *n* = 4), 0.25 (LOW, *n* = 2), 0.55 (MEDIUM, *n* = 2), or 0.85 (HIGH, *n* = 2) L/min for 100 min. The protocol for the 10 sows was repeated 8 times (2 times/day for 4 days). In each of the 8 repetitions, treatments assigned to the sows, the experimental unit, were switched randomly. The only 2 restrictions were that the same sow was never in the CONTROL treatment twice in the same day and all sows were exposed to each treatment at least once. The overall room temperature, relative humidity and dew point during the trial is presented on Table 1.

**Table 1:** Mean and standard deviation for temperature humidity and dew point during the trial^a^.

	**Temperature (°C)**	**Relative humidity (%)**	**Dew point (°C)**
Period 1	29.0 ± 2.5	75.9 ± 7.8	24.2 ± 1.9
Period 2	35.1 ± 0.4	68.5 ± 3.1	28.3 ± 1.0
Period 3	35.1 ± 0.4	68.4 ± 3.3	28.4 ± 1.0

a *Each value represented the mean of the 8 replications*.

### Data collection

#### Behavior

Behavior was recorded in real time over each experimental repetition using ceiling-mounted cameras (Panasonic WV-CP254H) attached to a digital video recording system (Geovision GV-1480). The behaviors defined in the ethogram (Table 2) were then extracted using continuous sampling by one trained individual using Observer XT 11 software (Noldus Information Technology, Leesburg, VA).

**Table 2:** Ethogram.

**Behavior**	**Definition**
Standing	The sow is standing, included kneeling
Sitting	The sow is sitting, contact between its bottom and the ground
Lying	The sow is either lying on her belly or laterally (accumulation of both lying behaviors below)
Sternal lie	The sow is lying on her sternum, udder completely or partially obscured under the sow
Lateral lie	The sow is lying on her side, both lines of teats not obscured
Drinker pressing/drinking	The snout of the sow is in contact with the drinker
Eating	The head of the sow is above the feeder, head down in the trough
Nursing	The sow is in a lateral recumbency and a minimum of three piglets are actively (head movements) touching the teat region

Each Period (1–3) was sampled entirely. The behaviors were split into two categories, the postures: stand, sit, and lie (sternally or laterally); the fundamental needs: drink, eat and the nursing activity. Postures were mutually exclusive behaviors, whereas fundamental needs were coded as “start-stop” behaviors. At all times, each sow was necessarily in one of the three listed postures. For each behavior listed, the total number of occurrences as well as the duration were measured. To standardize traits between repetitions, number of occurrences was expressed as number per hour, and duration as a percentage per hour.

Two sows of the 10 had missing data for the drinker pressing/drinking behavior: one for all the repetitions and the other one for the first five repetitions, due to partial obstruction of the camera's view. For three of the sows most distant from the camera, lying behavior was not defined as “sternally” or “laterally” because of the camera's brightness and contrast quality limits.

Drinking and eating bouts were determined using a bimodal Gaussian density curve to fit the behaviors' log-transformed intervals (35). The between-meal interval determined was 619 s, and the between drinking-bout interval was 2.29 s. The total number of drinking and eating bouts was corrected using this criterion.

#### Heart rate and heart rate variability

Heart rate monitors (Polar S810i, Polar Electro Öy, Kempele, Finland) were attached to six sows [see methodology in (36)], and set to record and store successive interbeat intervals (IBI) during each experimental repetition. The IBI data were then downloaded onto a computer via a Polar interface IR transfer (Polar Electro Öy, Kempele, Finland) and stored for later processing. Preliminary processing of the IBI data involved the visual and mathematical comparisons of individual beats with their neighboring beats to identify any anomalous or ectopic intervals. Spurious beats were assigned an error classification and carefully edited according to the recommendations for editing anomalous data published elsewhere (37).

Where possible, three sections of 512 beats (one per phase and approximately 4 min long) were extracted from each treatment for time and frequency domain analysis, together with non-linear (including geometric) analysis. Only sections with less than 0.5% error were used. Kubios HRV 2.1 software (38) was used to obtain heart rate variability (HRV) variables. The following 3-time domain variables were examined: (1) IBI mean (RR mean; RR is the interval between successive R peaks of the QRS complex of the Electrocardiogram wave). Interbeat interval mean provides general variability information (39); (2) root mean square of successive RR differences (RMSSD), which reflects the integrity of vagus nerve-mediated autonomic control of the heart; and (3) the standard deviation of all RR intervals of the dataset (SDNN), which is a good predictor of overall variability present at the time of recording.

Frequency domain analysis was done using a Fast Fourier Transformation (FFT) obtaining high (HF), and low frequency (LF) bands, expressed in normalized units (n.u.). Frequency bands widths (LF: 0.01–0.09 Hz; HF: 0.09–2.0 Hz) were assigned according to pig recommended ranges (40). The following 2 frequency domain variables were examined: (1) LF:HF ratio, also referred to as the Sympathetic Nervous System indicator (SNSI) is determined to reflect activity due to sympathetic activity and, (2) HF/total power, the Parasympathetic Nervous System indicator (PNSI), is used to enumerate vagal activity (39).

For geometric analysis, a Poincaré plot was plotted in Kubios and SD1 (short-term variability) and SD2 (long-term variability) was calculated. The following geometric variable was examined: (1) SD1:SD2 ratio, which is an indicator of sympathetic tone. Other non-linear analysis variables included: (1) recurrence rate, (2) sample entropy, and (3) Shannon entropy. These measures give an indication of the complexity within the IBI time series, with lower values indicative of increased sympathetic activity and decreased parasympathetic activity.

### Statistical analysis

Statistical analyses were performed with the software R (41). Because no difference between flow rates was obtained regarding all the behavioral and heart rate traits, the three flow rates groups were merged together to improve statistical power, and analyses were completed testing the effect of a cooling flow rate between 0.25 and 0.85 (COOLED) or no cooling flow rate (CONTROL). Effects of treatment on variables were estimated using repeated measures models with the function lmer from the R package “lme4.” For Period 1, the model included the treatment as fixed effect, and both the sow and the repetition being included as random effects. For Period 2, the Period 1 measurement was included in the previous model as a linear covariate. For Period 3, both Period 1 and Period 2 measurements were included as covariates. All data are reported as least-squares means and differences considered significant if *P* < 0.05. The bimodal Gaussian density curves to fit the eating and drinking's log-transformed intervals were drawn using the function mix from the R package “mixdist.”

## Results

### Behavior

There were no significant differences between treatments for all traits during Periods 1 and 2 (Table 3). During Period 3, CONTROL sows carried out more drinking/drinker pressing bouts and spent more time doing this behavior than COOLED sows (*P* < 0.001). CONTROL sows also had more bouts and total time spent in standing and sitting postures (*P* < 0.001). Although CONTROL sows had more total, sternal and lateral lying bouts than COOLED sows, the total time spent lying was less, especially lying laterally (*P* < 0.001). CONTROL sows were more active during the cooling Period 3, with more frequent change postures (*P* < 0.001). Eating behavior and nursing behavior were not impacted by the treatment (*P* > 0.1), with similar numbers of bouts and total time spent in each, between treatments.

**Table 3:** Effect of a cooling pad flow rate between 0.25 and 0.85 L/min (COOLED) or 0.0 L/min (CONTROL) on behavioral and heart rate traits before heating (Period 1), prior to cooling (Period 2), and during cooling (Period 3).^a^^,^^b^.

**Trait**^**3**^		**N**	**Period 1**		**Cooling effect**	**Period 2**		**Cooling effect**	**Period 3**		**Cooling effect**
			**CONTROL**	**COOLED**		**CONTROL**	**COOLED**		**CONTROL**	**COOLED**	
Drink	N	67	8.6 ± 2.6	11.4 ± 2.4	NS	10.1 ± 1.8	9.4 ± 1.7	NS	7.9 ± 1.0	2.7 ± 0.9	^***^
	%		5.7 ± 1.8	6.6 ± 1.7	NS	6.7 ± 1.4	6.8 ± 1.4	NS	4.4 ± 0.5	1.4 ± 0.42	^***^
Eat	N	80	0.49 ± 0.2	0.92 ± 0.2	NS	0.46 ± 0.2	0.30 ± 0.1	NS	0.33 ± 0.10	0.29 ± 0.09	NS
	%		0.85 ± 0.9	2.8 ± 0.8	NS	1.4 ± 0.7	0.95 ± 0.6	NS	1.8 ± 1.2	2.1 ± 1.1	NS
Nurse	N	56	1.49 ± 0.6	1.9 ± 0.5	NS	1.9 ± 0.2	1.7 ± 0.2	NS	2.0 ± 0.2	1.9 ± 0.1	NS
	%		0.14 ± 0.04	0.11 ± 0.03	NS	0.12 ± 0.03	0.16 ± 0.03	NS	0.18 ± 0.02	0.22 ± 0.02	NS
Stand	N	80	2.9 ± 0.6	3.2 ± 0.5	NS	2.4 ± 0.4	2.5 ± 0.4	NS	2.1 ± 0.2	0.72 ± 0.2	^***^
	%		11.0 ± 3.6	15.0 ± 3.3	NS	8.5 ± 1.8	7.4 ± 1.7	NS	6.6 ± 1.7	3.8 ± 1.6	^***^
Sit	N	80	5.7 ± 1.4	6.8 ± 1.4	NS	5.2 ± 1.0	5.0 ± 0.9	NS	4.5 ± 0.7	2.1 ± 0.7	^***^
	%		12.0 ± 3.5	12.0 ± 3.3	NS	15.0 ± 2.9	13.0 ± 2.7	NS	10.0 ± 1.2	4.0 ± 1.1	^***^
Lie	N	80	7.6 ± 1.3	8.1 ± 1.3	NS	5.7 ± 0.8	5.5 ± 0.8	NS	4.8 ± 0.6	2.3 ± 0.6	^***^
	%		77.0 ± 5.1	73.3 ± 4.9	NS	76.0 ± 2.9	80.0 ± 2.7	NS	83.0 ± 1.8	92.0 ± 1.7	^***^
Sternal lie	N	56	5.6 ± 1.8	7.5 ± 1.7	NS	5.4 ± 1.1	5.2 ± 1.1	NS	5.4 ± 0.8	2.5 ± 0.8	^***^
	%		29.0 ± 5.3	28.0 ± 4.4	NS	26.0 ± 4.2	19.0 ± 3.8	NS	19.0 ± 3.8	16.0 ± 3.5	NS
Lateral lie	N	56	3.6 ± 0.8	4.1 ± 0.8	NS	3.2 ± 0.3	2.9 ± 0.3	NS	2.6 ± 0.2	1.4 ± 0.2	^***^
	%		52.0 ± 9.2	40.0 ± 8.3	NS	48.0 ± 6.4	59.0 ± 5.7	NS	62.0 ± 5.6	75.0 ± 5.3	^***^
Posture changes, N		80	16.3 ± 2.8	18.1 ± 2.7	NS	13.3 ± 1.9	13.0 ± 1.9	NS	11.5 ± 1.6	5.1 ± 1.6	^***^
Mean_HR, bpm		14	114.8 ± 4.7	113.1 ± 4.4	NS	105.5 ± 3.5	112.8 ± 3.0	NS	119.0 ± 4.0	100.2 ± 3.4	^***^
SDNN		14	12.4 ± 3.0	13.9 ± 2.7	NS	12.7 ± 3.3	17.1 ± 2.9	NS	21.4 ± 2.5	17.5 ± 2.3	NS
RMSSD		14	4.2 ± 0.5	4.4 ± 0.5	NS	5.2 ± 0.9	4.7 ± 0.9	NS	5.2 ± 0.7	4.5 ± 0.7	NS
LF:HF		14	5.0 ± 1.6	7.0 ± 1.5	NS	6.9 ± 1.6	9.1 ± 1.4	NS	9.1 ± 4.7	6.7 ± 4.5	NS
HF/Total power		14	0.23 ± 0.05	0.15 ± 0.04	NS	0.20 ± 0.03	0.14 ± 0.02	NS	0.11 ± 0.06	0.25 ± 0.05	NS
SD1:SD2		14	0.21 ± 0.04	0.20 ± 0.04	NS	0.27 ± 0.06	0.18 ± 0.05	^*^	0.087 ± 0.04	0.20 ± 0.03	^**^
Recurrence rate		14	43.2 ± 2.2	45.9 ± 2.1	NS	41.3 ± 5.3	49.3 ± 4.9	NS	48.05 ± 5.1	49.5 ± 4.9	NS
Shannon entropy		14	3.6 ± 0.1	3.8 ± 0.1	NS	3.6 ± 0.2	3.9 ± 0.1	NS	4.0 ± 0.2	4.1 ± 0.2	NS
Sample entropy		14	0.99 ± 0.1	0.81 ± 0.1	NS	1.0 ± 0.1	0.79 ± 0.09	NS	0.61 ± 0.2	0.93 ± 0.2	^*^

a Statistical model formula for Period 1: Trait ~ treatment + Random(Sow) + Random (Repetition).

Period 2: Trait ~ treatment + measurement of Period 1 + Random(Sow) + Random (Repetition).

Period 3: Trait ~ treatment + measurement of Period 1 + measurement of Period 2 + Random(Sow) + Random (Repetition).

b Adjusted means ± SEM.

* P < 0.05;

** P < 0.01;

*** P < 0.001; NS: non-significant.

c *Traits: Mean_HR: mean heart rate; SDNN: standard deviation of all RR intervals; RMSSD: root mean square of successive RR differences; LF:HF: ratio between low (LF) and high frequency (HF) bands; HF/Total power: ratio between high frequency (HF) and total power; SD1:SD2: ratio between short-term variability (SD1) and long-term variability (SD2)*.

### Heart rate and heart rate variability

There were no significant differences regarding all traits during Periods 1 and 2, except for the SD1:SD ratio during Period 2 with CONTROL having higher values than COOLED sows (*P* < 0.05) (Table 3). During Period 3, CONTROL sows had higher mean heart rate than COOLED sows (*P* < 0.001), lower SD1:SD2 ratio (*P* < 0.01), as well as lower sample entropy (*P* < 0.05). The SDNN, the RMSSD, LF:HF, and HF/Total power ratios, recurrence rate, and Shannon entropy were not affected by treatment (*P* > 0.05).

## Discussion

The present study examined the effects of different cooling water flow rates through a cooling pad on lactating sows' behavioral and heart rate responses to acute heat stress. The effects on these sows' respiration rates, vaginal, skin, and rectal temperatures have been previously reported (32). These authors found beneficial effects of the cooling pad with a “dose-dependent” decrease in respiration rates and temperatures with increasing water flow rates from LOW to HIGH (from 0.25 to 0.85 L/min for 100 min) in comparison to a CONTROL flow rate (0.00 L/min). In the current study, we were unable to determine any differential effects of the LOW, MEDIUM or HIGH flow rates on the behavioral or heart rate parameters, but the use of cooling pads during an acute heat stress did provide beneficial effects for the COOLED sows.

Reduced feed intake has been reported in pigs to be one of the main physiological responses to prevent body temperature from increasing (5, 14, 42–44). However, this strategy appears to be temperature and/or time dependent. Decrease in feed intake is considered as a good indicator of discomfort in pigs (45). The beneficial effects of cooling systems on feeding behavior is not clear. Three studies have demonstrated an increase in time spent eating for both growing/finishing pigs exposed at room temperature above 25°C for several weeks (45), and lactating sows kept for 21 days at an ambient temperature between 20.8 and 29.5°C (27, 28). In contrast, de Oliveira et al. (46) have not reported any effect for cooled lactating sows exposed for a 28 days period at 25.7°C through the same floor cooling system. In the present study, feed was distributed three times per day right before the two Periods 1 and after the last Period 3 of the day. Sows were fed to target the mean *ad libitum* intakes and had a free access to the feeder anytime. Because of individual housing, they did not have any competition for feeding. Even if the distribution of fresh feed under neutral temperatures was attractive and initiated an important feed intake at that specific moment, eating bouts were well distributed all along the day. Therefore, no effect was expected because of the short 170–210 min acute heat stress on this minority behavior in a non-competitive system.

Heat stress can also result in an increase in drinking behavior due to discomfort and attempts to reduce the body temperature (47). The use of a floor cooling system has already been reported to decrease the frequency of drinker use (46) and the percentage of time spent drinking (25, 27). Similarly, in this study, cooled sows spent less time using the drinker and also had fewer bouts of drinker use. Two behaviors related to the drinker have been observed during live observations: actual drinking behavior and water spraying behavior. During this second behavior, sows used their snout to spray their face and body, which represents an alternative strategy to decrease their body temperature by evaporative cooling. Due to a lack of resolution in the video records, the distinction between both behaviors was not possible.

Finally, heat stress could also have repercussions on time spent nursing piglets. In the three previous studies mentioned using the same floor cooling system, one has not found any effect on nursing behavior (46), whereas the two others have found an increase in nursing time for cooled sows (27, 28). An increase in nursing behavior can be considered as an indicator of greater comfort for the sows (27). However, the definition of the nursing behavior can vary from one study to another and was not specified in those previous articles. Thus, comparison between studies is not possible. In this study, it was defined as the sow in lateral recumbency with at least three piglets actively touching the teat region. No beneficial effect of the cooling pad has been observed regardless of the flow rate considered. However, as with eating behavior, any effect of cooling is more likely to be seen with chronic heat stress over lactation (27, 28), rather than the acute heat stress protocol used in this study.

Another behavioral strategy to fight against heat stress is reducing general activity. When comparing a thermoneutral environment of 20°C to a heat stress environment of 28°C over a 6 days period, lactating sows have been observed to reduce standing and sitting postures from 18 to 11.6% of time (43, 48) and correspondingly increase lying postures, especially lying laterally (9). Moreover, non-lactating gilts maintained for 30 days in a hot environment of 30°C spent also more time lying laterally than lying sternally in comparison with neutral (21°C) and cold environments (15°C) (48). Lying laterally appears to be a strategical posture when pigs suffer from a heat stress. Huynh et al. (45) considered that lying is less energy consuming and the posture which results in more convective and conductive heat exchange, enabling the total heat load of the animal to decrease. The use of a cooling pad is therefore highly relevant, as it relies on taking advantage of a natural behavioral strategy. Indeed, sows usually spent more than 70% of the time lying down (27, 29, 49). Moreover, a comparison between cooling pads, drip cooling, and snout cooling carried out on penned gilts showed a preference for floor cooling systems with more spontaneous time spent on those devices (23). In lateral lying posture, the skin surface in contact with the floor is greater than in sternal lying, therefore maximizing the heat loss through conduction (9). Even if suitable statistical analyses were not possible in this study design, a decrease in average values of number of postures changes, time spent standing and sitting can be observed across the heat stress challenge from Period 1 to Period 3 in favor of lateral lying postures for both treatment groups. It confirms the use of a specific behavioral strategy of increased lying and reduced posture changes when subjected to an acute heat stress.

In cooling Period 3, COOLED sows had fewer bouts of standing, sitting, lying sternally and lying laterally than CONTROL sows, resulting in an overall decrease in posture changes. Regarding time spent in the different postures, COOLED sows spent less time standing and sitting in comparison to CONTROL, whereas they spent more time in both lying postures combined, especially lying laterally. Similar studies with floor cooling systems have also reported increased lying behavior for cooled sows (27, 29) and growing/finishing pigs (45, 50). A drip cooling system produced the same results of an increase in time spent lying and a decrease in posture changes (25). No difference was observed in the study of de Oliveira et al. (46), while their experimental design submitted the sows to a close range of temperatures and duration of heat stress. Silva et al. (28) observed more sitting and standing postures, as well as less time in lateral inactive lying postures. The authors concluded greater thermal comfort for cooled sows, spending less time inactive and showing less behavioral adaptation, but did not discuss the contrasting results to those seen in their previous study (27).

Heart rate variability represents a good non-invasive measure for assessing welfare in pigs (39). Literature about changes in heart rate measures in livestock during an acute heat stress challenge is missing. Mean heart rate was higher in CONTROL sows in Period 3 than COOLED sows. This rise in heart rate probably reflects an increase in sympathetic activity, as CONTROL sows also had higher SD1:SD2 ratio in Period 3. Decreased HRV and increased sympathetic activity in response to heat stress has been demonstrated in humans (51) and rats (52). No significant effects were observed on RMSSD and HF/total power ratio, two indicators of vagal activity, as well as on recurrence rate and Shannon entropy, two indicators of combined changes of both sympathetic and vagal activities. It may indicate that the increase in heart rate is only due to a stimulation of the sympathetic system with no changes in the vagal activity. CONTROL sows also showed a decrease in sample entropy in comparison to COOLED sows in Period 3, demonstrating less regularity of the heart rate signals. Disruption of vagal activity was already noticeable during Period 2, when the room temperature reached 35°C for an hour. CONTROL sows had higher SD1:SD2 ratio than COOLED sows on that period. It may indicate that SD1:SD2 ratio is a good early precursor of heat disturbance in lactating sows.

## Conclusion

Comparison between LOW, MEDIUM, and HIGH flow rates through a cooling pad in lactating sows under acute heat stress did not show any difference regarding behavioral traits of fundamental needs and postures, as well as heart rate parameters. However, the use of a cooling pad definitively confers thermal comfort to COOLED sows, that appeared quieter, stayed longer in lateral lying and had a low average heart rate compared to non-cooled sows. Behavioral and heart rate responses to heat stress can be effectively counteracted using water-cooled cooling pads from the lowest flow rate.

## Author contributions

SP was responsible for data extraction, analysis and interpretation, and was the principle author of the manuscript. FC was responsible for study design, data collection, and interpretation. AS was responsible for study conception, cooling pad development, data collection, and study coordination. JJ was responsible for study design, data analysis, and interpretation. RS was responsible for study conception, cooling pad design, development, and refinement. JM-F was responsible for study design, data collection, analysis and interpretation, and was a major author of the manuscript. All authors contributed to manuscript revision and have read and approved the final manuscript.

### Conflict of interest statement

The authors declare that the research was conducted in the absence of any commercial or financial relationships that could be construed as a potential conflict of interest.
